# Clinical significance and immune landscape analyses of the coagulation-related gene signatures in gastric cancer

**DOI:** 10.7150/jca.104221

**Published:** 2025-03-03

**Authors:** Yueming Yu, Dingwei Liu, Jun Xie, Zhou Feng, Xiaoping Huang, Hui Li, Yong Xie, Xiaojiang Zhou

**Affiliations:** Department of Gastroenterology, Digestive Disease Hospital, The First Affiliated Hospital, Jiangxi Medical College, Nanchang University, Jiangxi Province, China.

**Keywords:** gastric cancer, coagulation-related gene, tumor immune microenvironment, risk score, prognosis

## Abstract

Gastric cancer (GC) is one of the most common types of clinically malignant tumors and a global health challenge due to its high mortality and poor prognosis. The coagulation cascade is closely related to GC and plays a key role in the tumor immune microenvironment. However, the specific mechanisms by which coagulation-related genes involved in the occurrence and development of GC remains unclear. The data of GC patients and coagulation-related genes were obtained from the TCGA and the GSEA databases, respectively. After univariate Cox regression analysis, the non-negative matrix factorization method was used to identify coagulation-related molecular subtypes. GC patients were categorized into high-risk and low-risk score groups based on median risk scores, which included six genes (PCDHAC1, HABP2, GPC3, GFRA1, F5, and DKK1). There was a significant difference in survival between the two groups, and the predictive abilities for 1-, 3-, and 5-year survival were valid. Here, we demonstrated that coagulation-related gene signatures are valuable in predicting the survival of GC patients. Besides, the high- and low-risk grouping also better reflects the status of tumor mutation burden and the characteristics of tumor immune infiltration in GC, which provides a theoretical basis for individualized chemotherapy and immunotherapy for GC patients.

## Introduction

Gastric cancer (GC) is one of the most common malignant tumors worldwide, with the fifth highest incidence and mortality rate. Although its mortality rate has decreased in recent years, the prognosis for patients with advanced GC is still not optimistic^1^. In recent years, the treatment of GC has been richly developed in addition to traditional radical surgery^2^, ^3^. Emerging treatments such as neoadjuvant therapy, immunotherapy, and targeted therapy have provided additional options for patients^4^-^7^. In particular, the rapid development of immunotherapy has enabled patients with certain specific subtypes of GC to prolong their survival through new treatment pathways^8^. However, GC patients with MSI-H only account for about 10% of all patients, and the vast majority of patients with advanced GC still show insensitivity to immunotherapy, suggesting the need to explore new therapeutic drugs or targets for GC^9^, ^10^. In addition, even for GC patients with the same tumor stage, there are still huge differences in chemotherapy sensitivity and prognosis^11^. Therefore, biomarkers affecting the prognosis of GC need to be further explored.

Coagulation-related genes play important roles in cancer and other diseases. In recent years, more and more studies have revealed their potential mechanisms in tumorigenesis, progression, and metastasis, suggesting that these genes are not only involved in the process of blood coagulation. Still, they may also affect tumor progression and prognosis by regulating the tumor microenvironment^12^-^14^. In hepatocellular carcinoma, there is a significant correlation between coagulation-related genes and the tumor immune microenvironment (TIME), and the risk score can be used as a reliable prognostic biomarker^15^. In breast cancer, coagulation-related genes play a key role in the regulation of the tumor microenvironment, especially in predicting patient prognosis and response to chemotherapy^16^. The correlation between coagulation-related genes and the TIME has also been confirmed in colorectal cancer. The risk score can be used as a reliable prognostic biomarker for personalized regimens of chemotherapy and immunotherapy for colorectal cancer^17^. Although research has confirmed the correlation of coagulation-related genes with the prognosis of various tumors and the TIME, there is still a paucity of studies in the field of GC. Therefore, how coagulation-related genes contribute to the prognosis of GC patients and their correlation with the TIME needs to be further explored. This will not only help to identify potential prognostic biomarkers for GC but also may provide a decision basis for chemotherapy and immunotherapy in GC patients.

In this study, we performed the non-negative matrix factorization (NMF) cluster analysis of coagulation-related genes affecting the prognosis of GC to compare survival between groups, and the two groups with the most significant survival were compared differently. Prognosis-related differential genes were screened using the least absolute shrinkage and selection operator (LASSO) regression. Prognostic risk scores were calculated, and the prognostic prediction model was constructed. Next, GC patients were divided into two groups of high and low risk by the median of the risk score. The coagulation-related differential genes between the two groups were compared, and Gene Ontology (GO) and Kyoto Encyclopedia of Genes and Genomes (KEGG) enrichment analyses were performed. Then, the correlation between risk scores and clinicopathological features was analyzed; independent risk factors affecting prognosis were screened, a nomogram model for predicting prognosis was constructed, and model evaluation and validation were performed. In addition, the differences in tumor mutation burden (TMB) and TIME between the low-risk and high-risk groups were compared. Finally, the sensitivities of common chemotherapeutic drugs were compared between the low-risk and high-risk groups to predict the drugs that might be effective in treating patients with GC, and tumor immune single-cell analysis of prognostic genes was performed.

## Materials and methods

### Data acquisition

The mRNA sequencing (FPKM) data and corresponding clinical information of GC patients were downloaded from The Cancer Genome Atlas (TCGA; https://tcga-data.nci.nih.gov/tcga/). Detailed information on GC patients is listed in Supplementary Tables S1. The gene set “HALLMARK_INTERFERON_GAMMA_RESPONSE” was extracted from the molecular signature database of Gene Set Enrichment Analysis (GSEA) (https://www.gseamsigdb.org/). The symbols of coagulation-related genes are shown in Supplementary Table S2, which includes 293 genes.

### Molecular subtype identification based on the NMF algorithm

The NMF algorithm is a new clustering method. It can extricate sample classification from difficult positions where gene space is in high dimensionality and there are too few samples to further explore by using the NMF R package (PMID: 23261450).

### Identification of coagulation-related genes

Significant differential expression of coagulation-related genes was identified by the “limma” package in R software with |log2FC|≥1 and FDR<0.05. There were 141 genes identified in the intersection of the different expression genes shown in Supplementary Table S3.

### Construction and validation of the risk model based on coagulation-related genes

First, all GC patients were divided into training and validation groups at a 7:3 ratio by “caret” package randomly. Then, we used the least absolute shrinkage and selection operator (LASSO) analysis by the “glmnet” package based on these 141 differentially expressed coagulation-related genes. Thirdly, we calculated the risk score for every GC patient. All the patients were divided into high-risk and low-risk subgroups by the median value according to the risk score. Kaplan-Meier (KM) analysis was used to evaluate the prognostic performance of coagulation-related gene signatures. We used the “timeROC” package to plot time-dependent receiver operating characteristic (ROC) curves.

### Nomogram model construction and validation

To assess whether the risk score generated from the coagulation-related genes could serve as an independent prognostic factor, we performed univariate and multivariate Cox regression analyses. The results were presented as forest plots. Decision Curve Analysis (DCA) was employed to quantify the net benefit of using the risk score for clinical decision-making across a range of threshold probabilities. By comparing the outcomes of patients who were stratified based on the risk score with those of patients who were not, DCA provides a graphical representation of how much benefit a model offers when implemented in clinical practice. We also plotted a Clinical Impact Curve (CIC), which shows the predicted number of high-risk patients and the number of true positives at various thresholds. The CIC complements the DCA by offering a more detailed understanding of how the risk score might influence patient management on a population level. Next, we constructed a nomogram that integrates the risk score along with other key clinical variables such as age and cancer stage to predict 1-, 3-, and 5-year survival probabilities for GC patients. The calibration curves were performed to evaluate the predictive accuracy of the nomogram.

### Immune cell infiltration analysis

We used the CIBERSORT algorithm to evaluate the infiltration levels of 22 tumor-infiltrating immune cells. We calculated the scores of the immune microenvironment by the ESTIMATE algorithm and researched the differences in the activity of immune infiltrating cells and 30 immune checkpoints of subgroups, using the "estimate," "GSVA," and “GSEABase” packages. Seven steps that occur in the tumor microenvironment are characteristic of anticancer immune responses. We analyzed the anticancer immune status and the extent of tumor-infiltrating immune cells, as well as differences in the activity scores across seven steps between the high- and low-risk groups through Tracking Tumor Immunophenotype (TIP; http://biocc.hrbmu.edu.cn/TIP/).

### Analysis of somatic mutation

The VarScan platform data from the TCGA-STAD cohort were used to analyze the somatic mutation data for each patient. Next, we used the “maptools” package to visualize the mutations between the two risk groups. Moreover, we analyzed the correlation between the TMB and the prognostic signature.

### Enrichment analysis of gene set

Spearman correlation analysis was performed to analyze the correlations of the six key prognostic genes. The GeneMANIA (http://www.genemania.org) website was used to construct a protein-protein interaction (PPI) network for the six key prognostic genes. GO and KEGG pathway enrichment analyses were performed to analyze the different expression genes between the high- and low-risk groups.

### Analysis of chemotherapy drug susceptibility

The Genomics of Drug Sensitivity in Cancer (GSDC) database (https://www.cancerrxgene.org/) was used to evaluate the chemotherapeutic drug response of the STAD patients (PMID: 23180760). We used the “pRRophetic” package to calculate the half-maximal inhibitory concentration (IC50) and assessed the STAD patient response to common chemotherapeutic agents. Furthermore, we used the Connectivity Map (cMap; https://portals.broadinstitute.org/cmap/) database to predict drugs based on the prognostic signature. Enrichment scores ranged from -1 to 0, and p < 0.05 was considered to indicate a potential candidate compound. We obtained the 3D structures of these compounds from the PubChem database (https://pubchem.ncbi.nlm.nih.gov/).

### Tumor immune single cell analysis of prognostic coagulation-related genes

Immune cell type clustering in the TIME of gastric cancer patients was analyzed using the Tumor Immune Single-cell Hub (TISCH) online website (http://tisch.comp-genomics.org). Prognostic coagulation-related genes were then compared for differences in immune cell content in the immune microenvironment.

### Statistical analysis

All the statistical analyses and plotting were performed by R software (version 4.0.5, https://www.r-project.org/). Univariate and multivariate Cox regression analyses were utilized to select independent prognostic factors for STAD patients. Spearman's correlation was used for correlation analysis. All P values were two-sided, and the results were considered statistically significant when P values were less than 0.05.

## Results

### Molecular subtyping of coagulation-related genes

**Figure 1:**
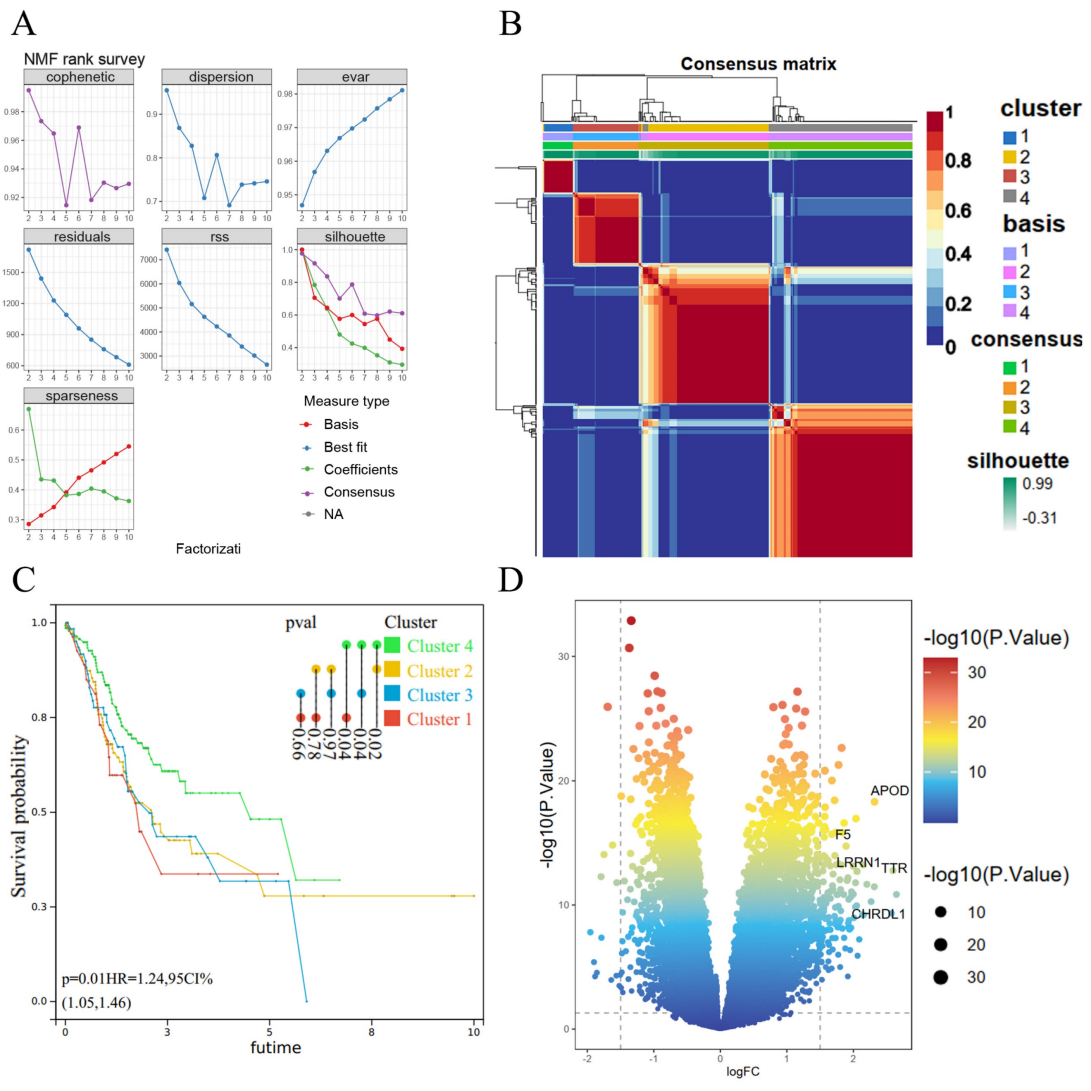
NMF algorithm for molecular subtype identification and coagulation-related differential genes. (A) Screening of cluster groupings; (B) Subgroup consistency analysis; (C) Subgroup survival analysis; (D) Volcano plots.

We subjected 293 coagulation-related genes to COX regression and found that 36 genes were associated with GC prognosis. These 36 prognosis-related genes were subjected to clustering analysis by the NMF algorithm and had an optimal classification when the subtype was 4 (Figure 1A). Subgroup correlation analysis revealed a high degree of consistency among subgroups (Figure 1B). We further performed survival analysis for each subgroup and found a significant correlation between Cluster 4 and Cluster 1, Cluster 2, and Cluster 3; among them, the difference between Cluster 4 and Cluster 1 was the most significant for GC patients, with a p-value of 0.02 (Figure 1C). Therefore, we compared the coagulation-related genes of GC patients in cluster 4 and cluster 1 as a subgroup and obtained 141 differential genes (Figure 1D).

### Construction and validation of prognostic risk models for coagulation-related genes

**Figure 2:**
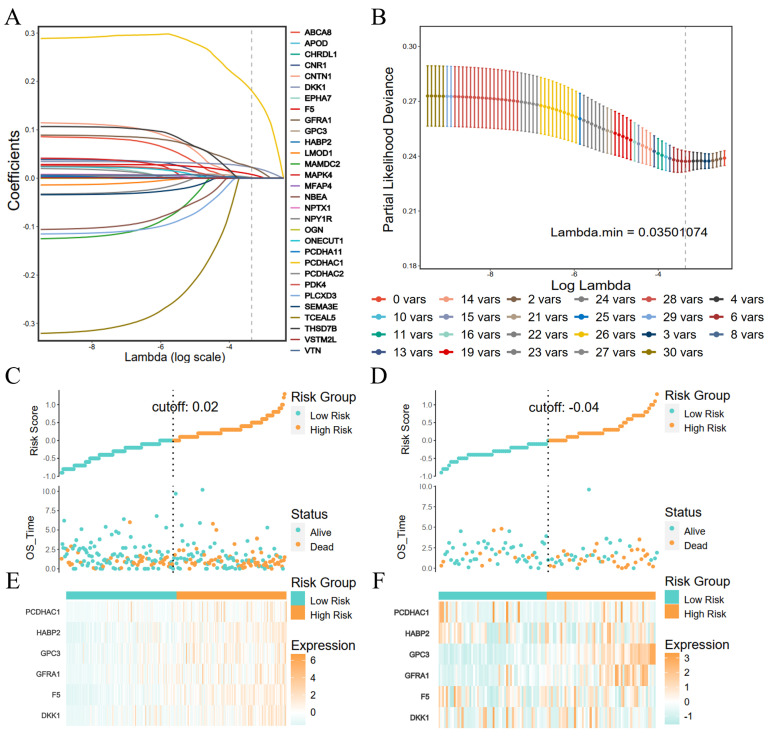
LASSO regression feature screening and risk score grouping. (A) LASSO regression coefficients for each feature; (B) Number of LASSO regression features screened; (C) Risk scores and survival in the training group; (D) Risk scores and survival in the validation group; (E) Heat map of risk scores and coagulation-related genes in the training group; (F) Heat map of risk scores and coagulation-related genes in the validation group.

**Figure 3:**
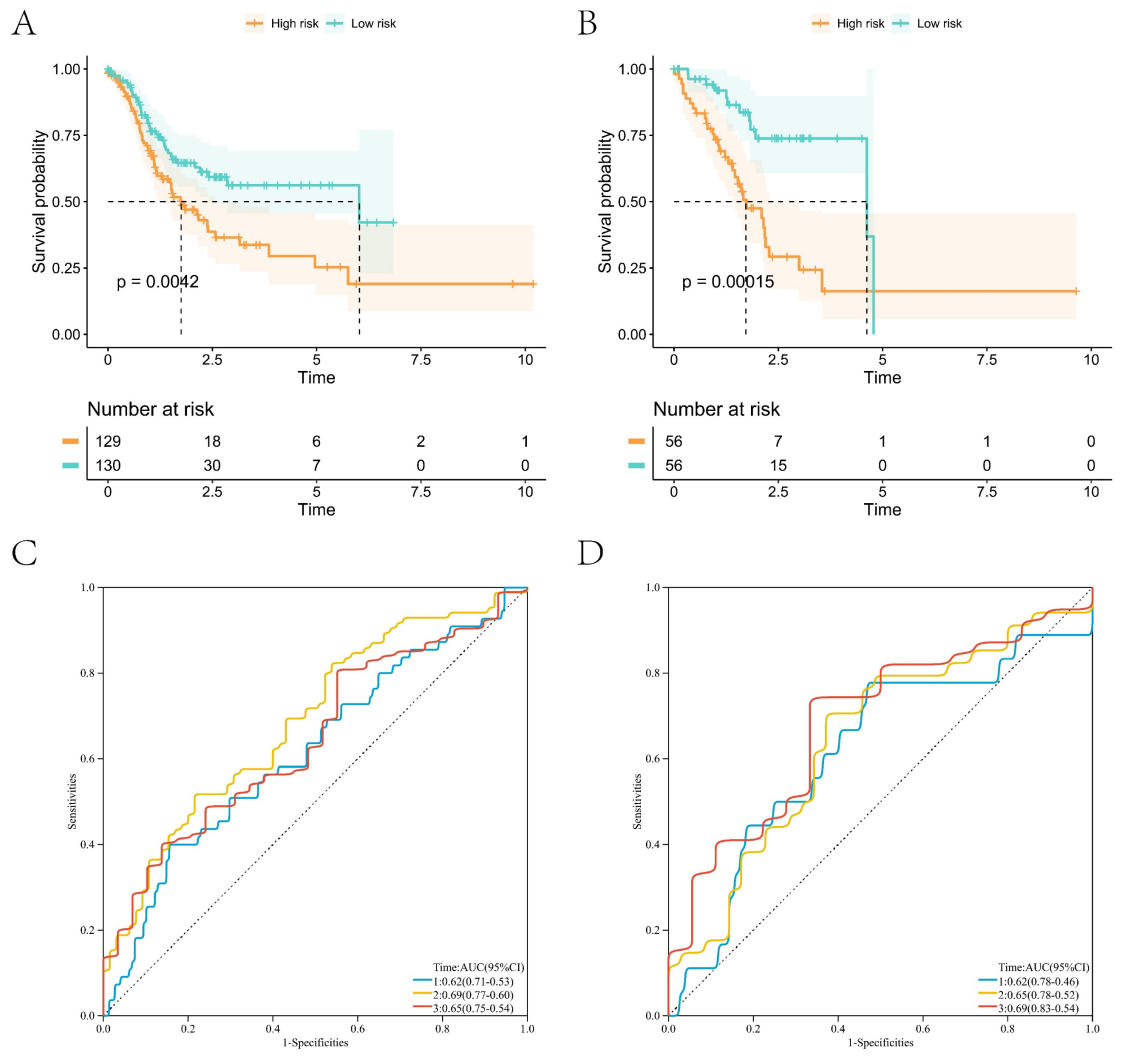
High- and low-risk group survival analyses with ROC curves for survival prediction models. (A) Survival analysis of the high- and low-risk groups in the training group; (B) Survival analysis of the high- and low-risk groups in the validation group; (C) ROC curves of the survival prediction model in the training group; (D) ROC curves of the survival prediction model in the validation group.

The Lasso regression was performed with the coagulation-related differential genes in the training group, and the regression coefficients of the corresponding coagulation-related genes decreased with increasing λ values (Figure 2A). Six coagulation-related genes affecting prognosis were finally screened out, and the risk scores were calculated (Figure 2B). Among them, DKK1, F5, GPC3, HABP2, and PCDHAC1 prognostic genes were highly expressed in the GC tissues, and GFRA1 was lowly expressed in GC tissues (Supplementary Figure S1). Patients were divided into a high-risk score group and a low-risk score group by the median risk score. In the training group, the low-risk score group had better survival than the high-risk score group; the six prognostic genes in the low-risk score group had lower expression than those in the high-risk score group (Figure 2C). In the validation group, the low-risk scoring group still had higher survival, and prognostic genes had lower expression (Figure 2D). Survival analysis found that patients in the low-score group had a better prognosis in the training and validation groups, and the difference was statistically significant (Figure 3A, B). We further plotted ROC curves predicting the overall survival of patients using risk scores. In the training group, the AUC values for predicting overall survival of GC patients at 1, 3, and 5 years were 0.62, 0.69, and 0.65, respectively (Figure 3C); in the validation group, the AUC values for predicting overall survival of GC patients at 1, 3, and 5 years were 0.62, 0.65, and 0.69, respectively (Figure 3D).

### Clinical correlation analysis of risk scores and construction of prognostic nomogram

**Figure 4:**
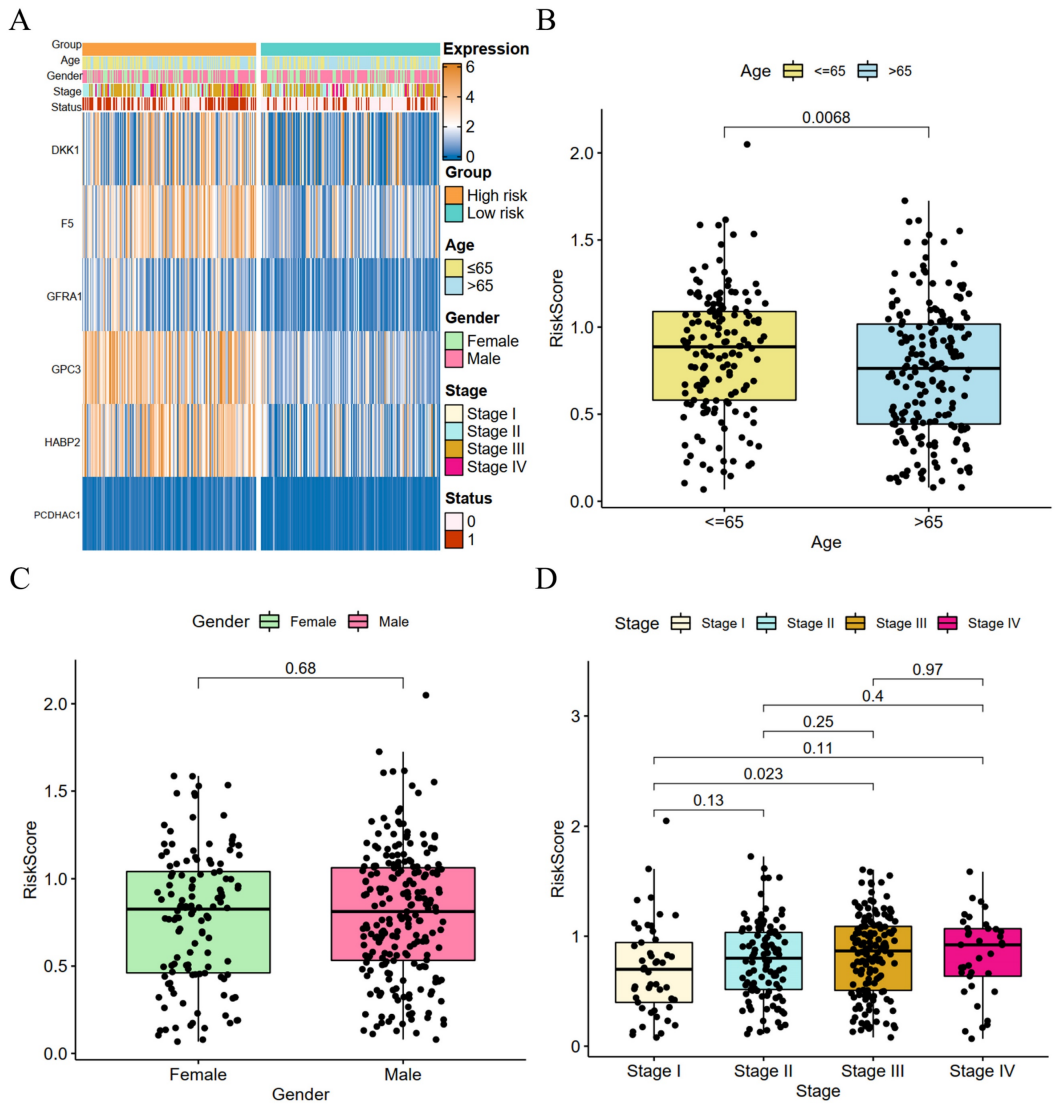
Risk score and clinical correlation analysis. (A) Heat map of high- and low-risk score subgroups with clinicopathologic features and coagulation-related genes; (B) Correlation between age and risk score (C) Correlation between gender and risk score; (D) Correlation between tumor stage and risk score.

We performed a clinical correlation analysis of risk scores (Figure 4A), and patients over 65 years old with advanced GC and a high-risk score had a greater fatality rate. There was no significant difference in the gender of the two groups. The coagulation-related genes of DKK1, F5, GPC3, GFRA1, and HABP2 were highly expressed in the high-risk group, whereas there was no significant difference between the two groups in PCDHAC1. GC patients aged 65 years or older had higher risk parity scores than GC patients younger than 65 years, and the difference was statistically significant (Figure 4B), whereas there was no statistically significant difference between the risk scores of male and female GC patients (Figure 4C). The risk scores showed a positive correlation with the GC stage, and there was a significant difference between the risk scores of patients with stage I GC and those with stage III GC (Figure 4D).

**Figure 5:**
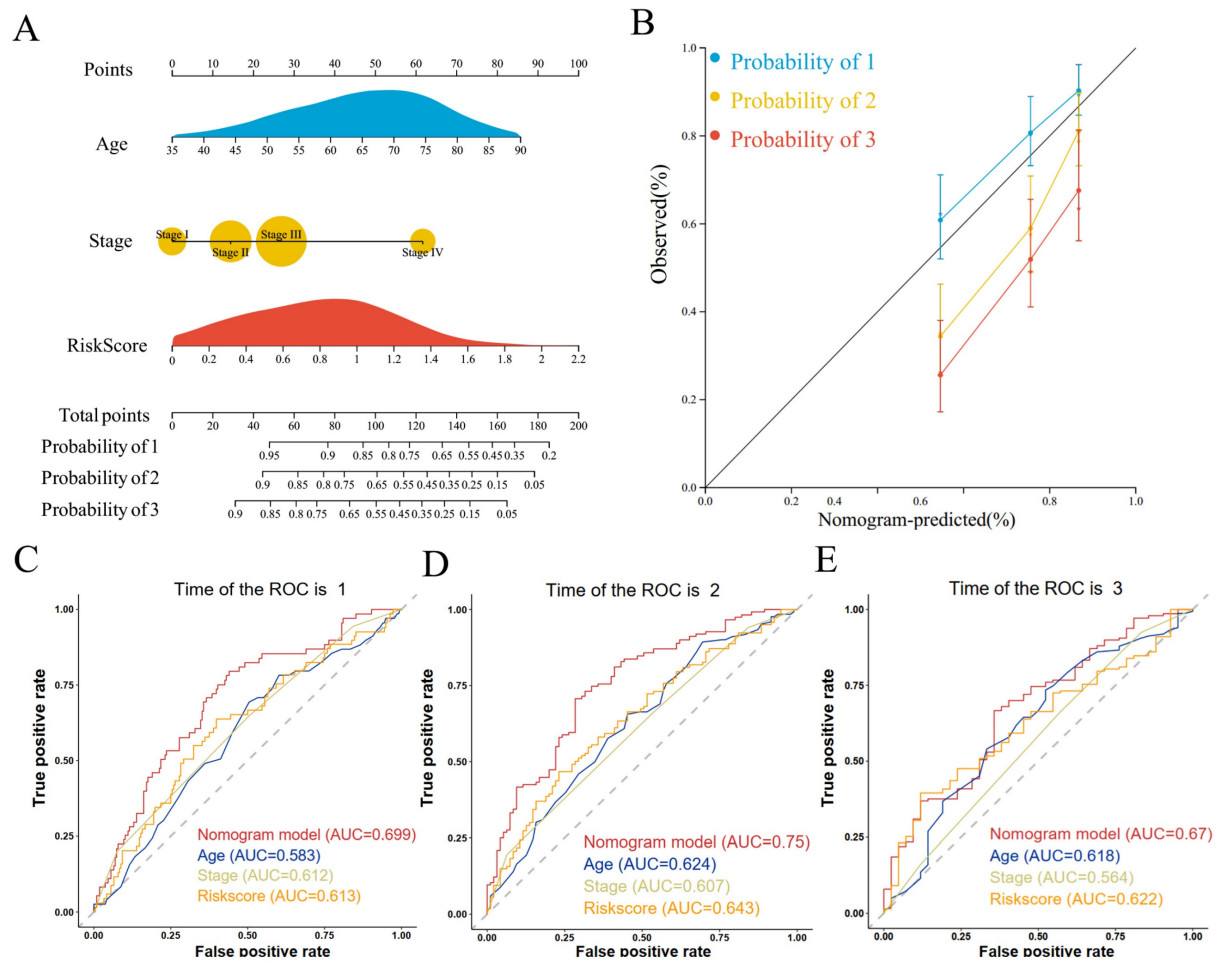
Construction and evaluation of prognostic prediction models. (A) Nomogram prediction model; (B) Calibration curves; (C) ROC curves for 1-, 3-, and 5-year survival.

We performed univariate and multivariate COX regression of age, gender, tumor stage, and risk score and found that age, tumor stage, and risk score were independent risk factors affecting prognosis in both univariate and multivariate COX regressions (Supplementary Figure S2A, B). Therefore, we constructed a nomogram for predicting the prognosis of GC based on age, tumor stage, and risk score (Figure 5A). The calibration curves showed that the predictive ability of 1- and 3-year overall survival was better fitted to the ideal curve, whereas the predictive ability of 5-year overall survival was poorer than that of the 1- and 3-year overall survival plots (Figure 5B). The nomogram had an AUC value of 0.699 for the 1-year overall survival prediction model (Figure 5C), 0.750 for the 3-year overall survival prediction model (Figure 5C), and 0.670 for the 5-year overall survival prediction model (Figure 5C). The nomogram had higher AUC values than the prognostic model constructed from age, tumor stage, and risk score individually. This suggests that the prognostic model constructed from the nomogram constructed from age, tumor stage, and risk score has higher accuracy. Decision curve analysis showed that the net benefit of the nomogram prognostic model was also higher than that of the prognostic model constructed from age, tumor stage, and risk score each (Supplementary Figure S2C). The results of the clinical impact curve showed that the predicted GC deaths gradually approached and tended to overlap with the actual GC deaths when the threshold value exceeded 0.4 (Supplementary Figure S2D).

### Correlation of risk scores with TMB and TIME

**Figure 6:**
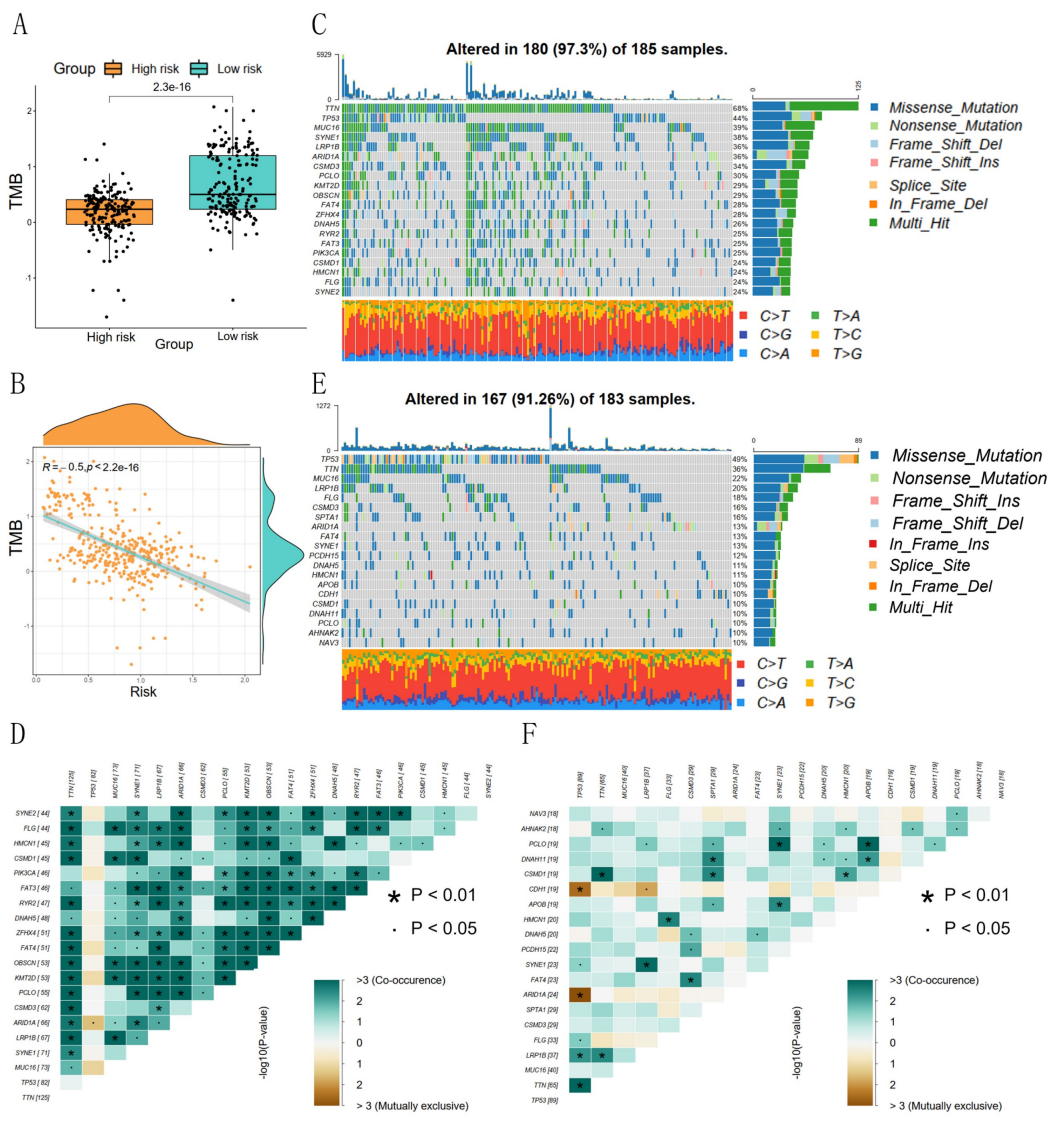
Correlation between risk score and tumor mutation burden. (A) Differences in TMB between high- and low-risk groups; (B) Correlation between risk scores and TMB; (C, D) Waterfall plots of TMB and co-occurrence of mutated genes in low-risk groups; (E, F) Waterfall plots of TMB and co-occurrence of mutated genes in high-risk groups.

The prognostic risk score and TMB correlation analysis showed that the low-risk group had a higher number of base mutations than the high-risk group (Figure 6A), and the risk score was significantly negatively correlated with the tumor mutation burden (Figure 6B). The waterfall plot showed that the low-risk group was mainly characterized by mutations in TTN, TP53, MUC16, SYNE1, and LRP1B, and the mutated base pairs were mainly C-G and T-A (Figure 6C). The high-risk group was mainly characterized by mutations in TP53, TTN, MUC16, LRP1B, and FLG, and the mutated base pairs were mainly C-G (Figure 6E). There was a significant co-occurrence of mutated genes in the low-risk group, which indicated that the number of mutated genes was higher in the same GC patients in the low-risk group (Figure 6D). There was a weak co-occurrence of mutated genes in the high-risk group, which indicated that the number of mutated genes in the same GC patients in the high-risk group was lower (Figure 6F).

**Figure 7:**
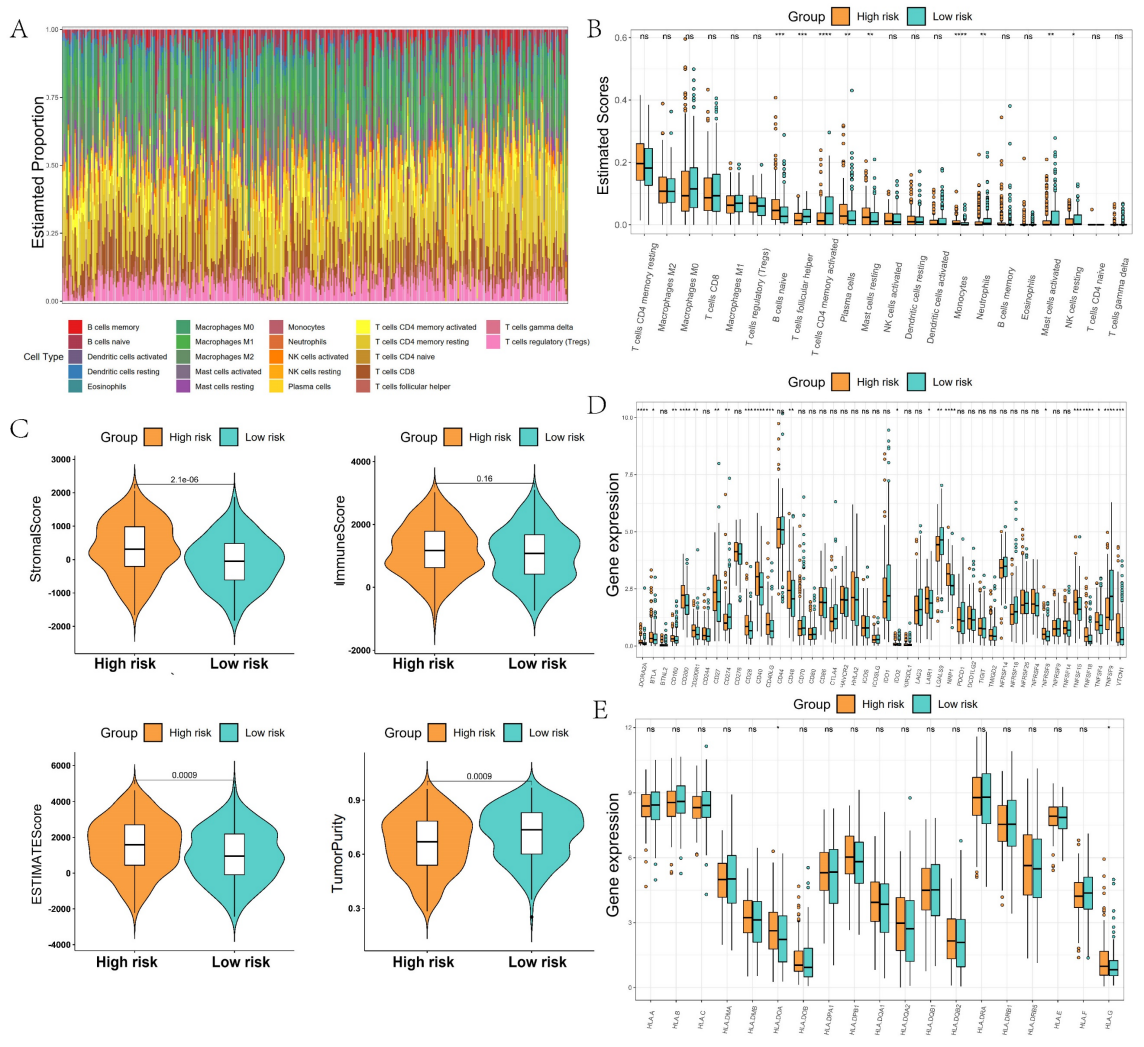
Comparison of the tumor immune microenvironment in high- and low-risk groups. (A) Proportion of immune cells constituting the tumor microenvironment; (B) Comparison of differences in immune cell content between high- and low-risk groups; (C) Comparison of differences in tumor microenvironment scores between high- and low-risk groups; (D) Comparison of differences in immune checkpoints between high- and low-risk groups; (E) Comparison of differences in human leukocyte antigens between high- and low-risk groups.

We further analyzed the immune microenvironment of GC, which was mainly composed of B cells, M0/M1/M2 macrophages, CD4 T cells, and CD8 T cells (Figure 7A). The composition ratio of immune cells had large differences among different GC patients. The content of naive B cells, plasma cells, and mast cells resting were significantly higher in the high-risk group than in the low-risk group, whereas the content of T cells follicular helper and T cells CD4 memory activated were significantly lower in the high-risk group than in the low-risk group (Figure 7B). The TIME score and stroma score of patients in the high-risk group were significantly higher than those in the low-score group. The tumor purity of GC patients in the high-risk group was significantly lower than that of the low-risk group, which indicated that GC patients in the low-risk group might have more immune cell infiltration (Figure 7C). Immune checkpoint difference analysis showed significant differences in checkpoints such as ADORA2A, CD200, CD28, and CD40 in the high-risk and low-risk groups, which may predict different responses to immunotherapy in the high- and low-risk groups (Figure 7D). Human leukocyte antigen analysis showed that the expression of HLA.DOA and HLA.G was significantly higher in the high-risk group than in the low-scoring group (Figure 7E).

### GO and KEGG enrichment analysis of coagulation-related genes

**Figure 8:**
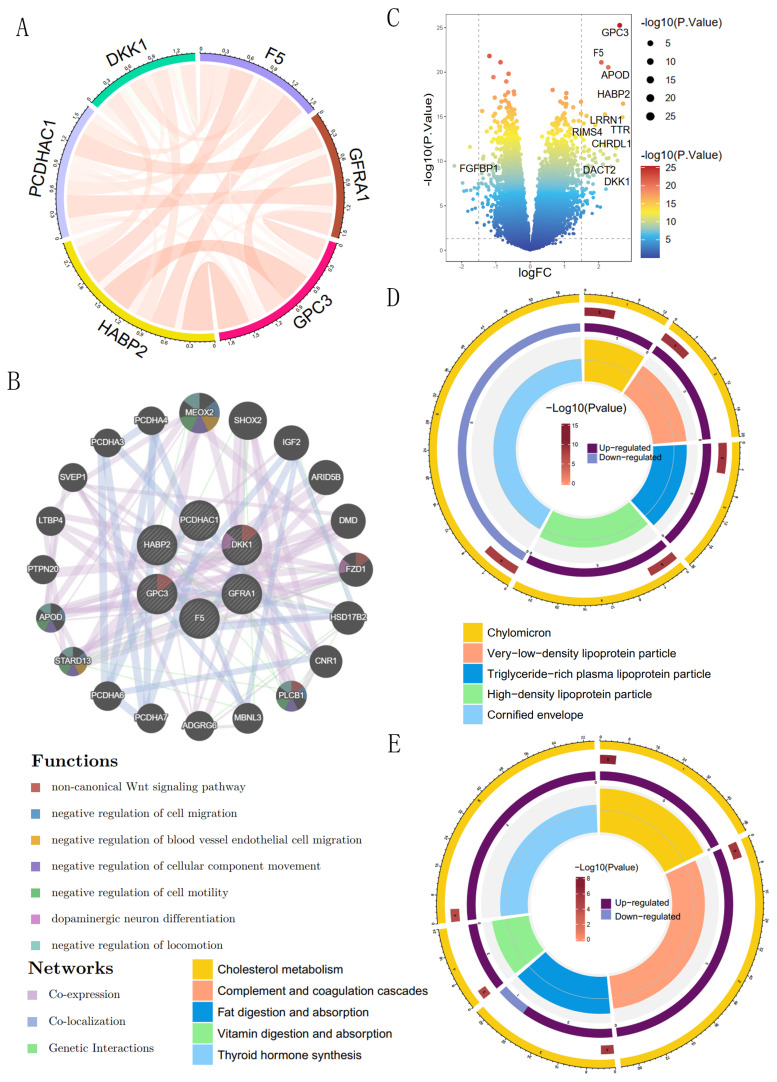
GO and KEGG enrichment analysis of coagulation-related differential genes. (A) Circle plot of prognostic coagulation-related genes; (B) Volcano plot comparing the differences in coagulation-related genes between high- and low-risk groups; (C) Protein interactions analysis of prognostic coagulation-related genes; (D) Circle plot of GO enrichment analysis; (E) Circle plot of KEGG enrichment analysis.

We analyzed the interrelationships among six coagulation-related genes affecting prognosis and found significant interactions among DKK1, F5, GPC3, HABP2, PCDHAC1, and GFRA1 (Figure 8A). The six prognostic genes were further analyzed for possible other interacting genes; the results showed that MEOX2, APOD, STARD13, PLCB1, and FZD1 had interactions, co-expression, and co-localization with the prognostic genes (Figure 8B). Expression of the genes, such as MEOX2, APOD, and other genes, may activate the Wnt signaling pathway, the regulation of cell migration, the regulation of blood vessel endothelial cell migration, the regulation of cell motility, and other signaling pathways to promote the development of GC. The 86 differential genes were screened according to the high- and low-risk scores; among them, GPC3 and F5 were significantly overexpressed in the high-risk group, and FGFBP1 was significantly overexpressed in the low-risk group (Figure 8C). GO enrichment analysis showed that prognostic coagulation-related genes might influence the chylomicrons, very-low-density lipoprotein particle, and high-density lipoprotein particle in exerting their biological functions (Figure 8D). KEGG enrichment analysis showed that prognostic coagulation-related genes might be involved in the regulation of pathways such as cholesterol metabolism, complement and coagulation cascades, fat digestion and absorption, vitamin digestion and absorption, and thyroid hormone synthesis (Figure 8E).

### Chemotherapy drug screening and tumor immune single cell analysis

**Figure 9:**
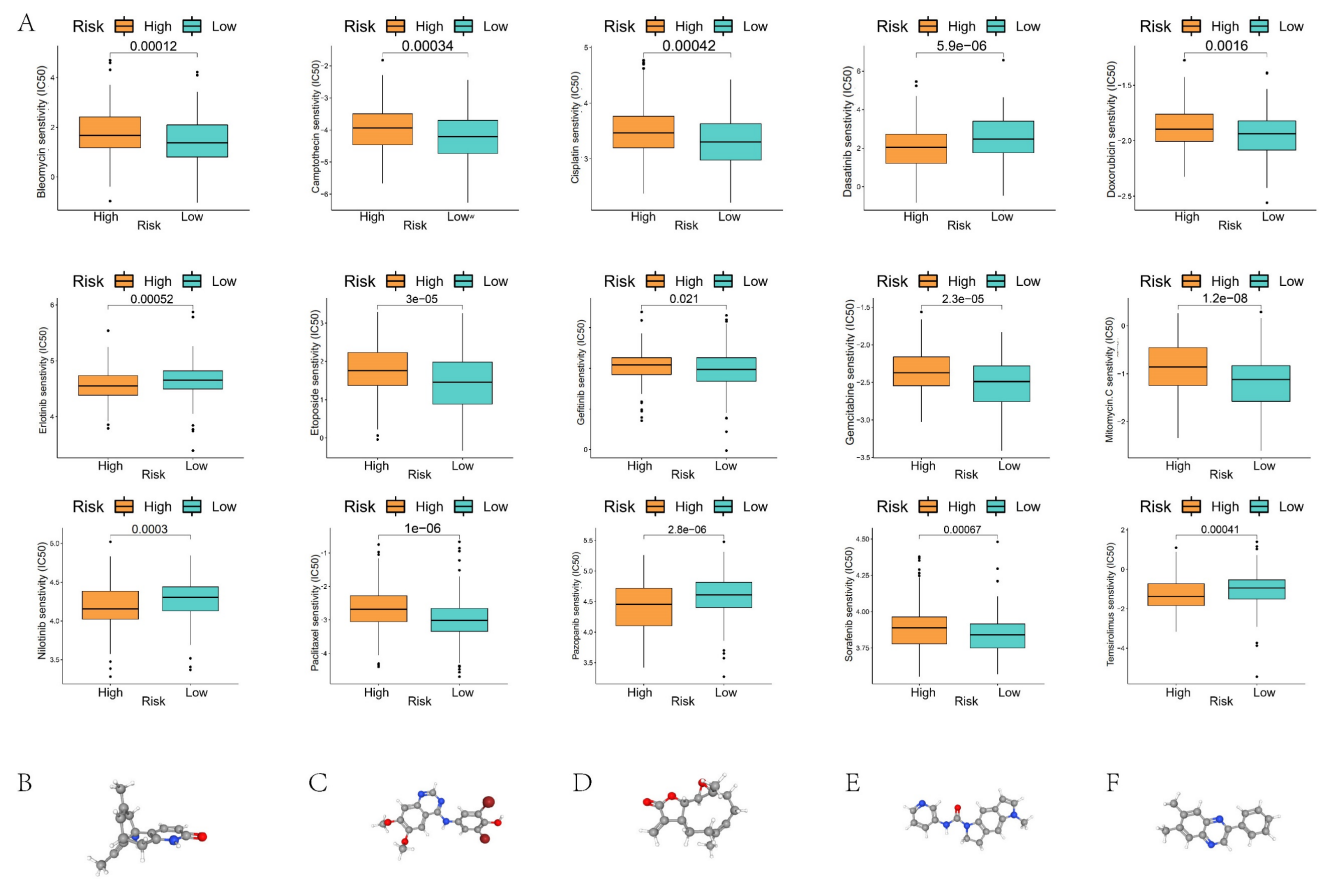
Sensitivity analysis of chemotherapeutic drugs in GC patients of high- and low-risk groups. (A) Comparison of IC50 values of chemotherapeutic drugs in GC patients of high- and low-risk groups; (B-F) Potential drug molecular structures and pharmacological targets affecting the prognosis of GC.

Chemotherapy drug sensitivity analysis showed significant differences in the half-maximal inhibitory concentrations (IC50) of 15 common chemotherapy drugs, including Bleomycin, Camptothecin, Cisplatin, and Dasatinib, in the high- and low-risk groups (Figure 9A). We used cMap to screen potential pharmacological targets affecting prognostic genes, and the results showed that Huperzine-A, KIN001-055, parthenolide, SB-206553, and tyrphostin-AG-1295 might be effective drugs affecting the prognosis of GC patients in the high- and low-risk groups (Figure 9B).

**Figure 10:**
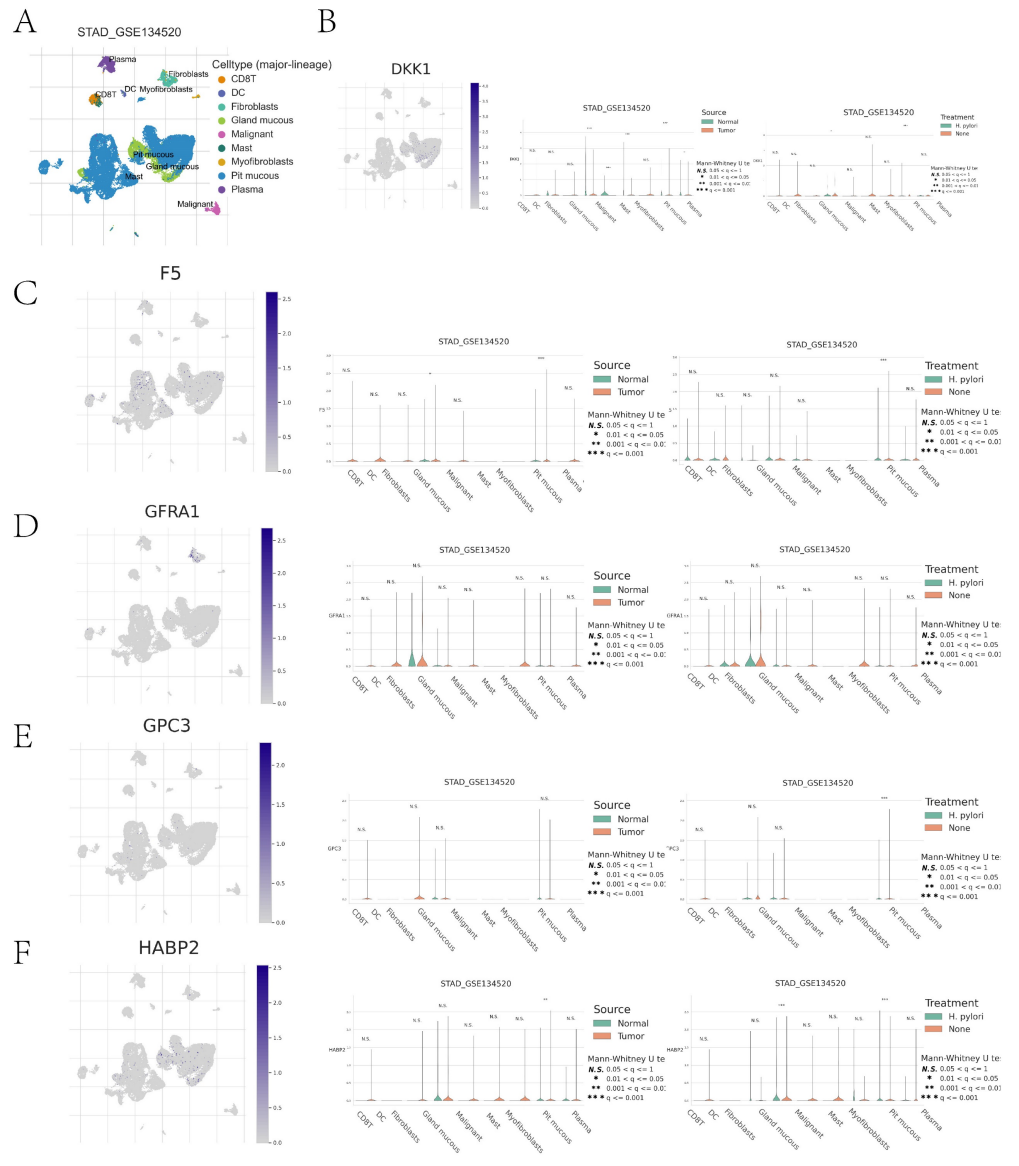
Cell clustering typing of tumor microenvironment and comparison of differences between high- and low-risk groups. (A) Cell clustering typing of tumor microenvironment; (B-F) Comparison of cell type differences in tumor microenvironment between high- and low-risk groups for DKK1, F5, GFRA1, GPC3, GPC3 in GC tissues versus normal tissues, and *H. pylori*-infected versus non-infected GC tissues.

Immune cell type clustering and annotation analysis of the GSE134520 dataset of GC patients were performed using the TISCH online data site. The tumor microenvironment of the GSE134520 dataset was dominated by nine cellular components, including pit mucous, gland mucous, plasma, fibroblasts, and CD8 T cells (Figure 10A). We further analyzed the distribution and expression of prognostic genes in different cell types. DKK1 was significantly expressed in pit mucous mainly in GC tissues; in GC tissues and normal tissues, the expression of DKK1 in gland mucous, malignant, mast, and pit mucous was significantly different; in *Helicobacter pylori* (*H. pylori*) infected and non-infected GC tissues, the expression of DKK1 in pit mucous showed significant differences (Figure 10B). F5 was also significantly expressed in pit mucous, mainly in GC tissues; in GC tissues and normal tissues, there was a significant difference in the expression of F5 in pit mucous; in *H. pylori*-infected and non-infected GC tissues, there was a significant difference in the expression of F5 in pit mucous (Figure 10C). GFRA1 was significantly expressed in GC tissues, mainly in fibroblasts, whereas there was no statistically significant difference in the expression of GFRA1 in nine cellular components, including pit mucous, gland mucous, and plasma, between *H. pylori*-infected and non-infected GC tissues in GC tissues and normal tissues (Fig. 10D). GPC3 was significantly expressed in pit mucous, mainly in GC tissues, and there was no statistically significant difference in the expression of GPC3 in pit mucous in GC tissues and normal tissues (Fig. 10D). In GC tissues and normal tissues, there was no statistically significant difference in the expression of GPC3 in pit mucous, gland mucous, plasma, and other 9 cellular components; in *H. pylori*-infected and non-infected GC tissues, there was a significant difference in the expression of GPC3 in pit mucous (Figure 10E). HABP2 was significantly expressed in pit mucous, mainly in GC tissues; in GC tissues and normal tissues, there was no statistically significant difference in the expression of HABP2 in pit mucous and plasma (Figure 10E). The expression of HABP2 in pit mucous was significantly different in both GC tissues and normal tissues, *H. pylori*-infected and non-infected GC tissues (Figure 10F).

## Discussion

Patients with malignant tumors often suffer from dysregulation of the coagulation system and systemic hypercoagulability, which is closely related to the aggressiveness and metastasis of the tumor^18^-^20^. Patients with malignant tumors often have an increased risk of thrombosis, especially venous thromboembolism, and venous thrombosis is one of the important causes of death in cancer patients. In patients with high-risk tumors such as GC, the incidence of venous thromboembolism (VTE) is as high as 20%, which not only affects the quality of life of patients but also becomes one of the important causes of death in cancer patients^21^, ^22^. Studies have shown that some coagulation indices are closely related to the prognosis of patients. D-dimer levels in breast cancer patients were closely related to tumor metastasis and prognosis, and breast cancer patients with hypercoagulable states had higher mortality rates^23^, ^24^. In addition, with the spread of immunotherapy, more and more patients have developed abnormalities in the coagulation system, and such abnormalities were closely associated with treatment effects and prognosis^25^, ^26^.

Because of this, our study confirmed that some coagulation-related factors can be used as biomarkers for the prognosis of GC patients and we further explored the effects of these factors on the microenvironment of immune infiltration in GC. Our findings will provide new ideas and rationale for improving the prognostic assessment and personalized immunotherapy for GC patients.

In our study, by Lasso regression analysis, we screened six coagulation factors associated with the prognosis of GC patients and constructed a prognostic prediction model. The results of ROC curve analysis showed that these screened coagulation-related genes had certain predictive abilities and could effectively distinguish GC patients at different prognostic risks. In addition, existing studies have provided support for the clinical application of coagulation factors. For example, Li *et al.*^14^ constructed a prognostic prediction model for coagulation-related genes in lung cancer patients based on the TCGA database, and the results showed that their AUC values for 1, 3, and 5 years were 0.601, 0.597, and 0.615, respectively, suggesting that the model has some practical value in predicting the prognosis of lung cancer. Similarly, Jin *et al.*^17^ constructed a prognostic prediction model for colorectal cancer in the TCGA database, with AUC values of 0.754, 0.756, and 0.755 at 1, 3, and 5 years, respectively, which further confirmed the importance of coagulation factors in tumor prognostic assessment. Although these findings support the potential role of coagulation-related genes in predicting tumor prognosis, further studies are needed to validate coagulation factors as biomarkers of GC prognosis.

We performed GO and KEGG enrichment analysis of coagulation-related differential genes and showed that the enriched pathways were mainly focused on lipid metabolism, especially cholesterol metabolism, fat digestion and absorption, and thyroid hormone synthesis. Among them, cholesterol metabolism plays a key role in tumorigenesis and tumor development^27^-^29^. Previous studies have shown that pancreatic cancer growth was closely associated with aberrant cholesterol metabolism. Zheng *et al.*'s^30^ study pointed out that in Wnt-dependent pancreatic ductal adenocarcinoma, cholesterol promotes tumor growth through the fzd5-mediated Wnt/β-catenin signaling pathway. In addition, the results of Tang *et al.*^31^ showed that cholesterol metabolism-related genes were effective in predicting the prognosis and immunotherapy response of GC. The results of Yue *et al.*^32^ further suggested that the long-chain non-coding RNA Linc01711 was involved in the progression of GC through histone modification-mediated reprogramming of cholesterol metabolism. Therefore, future evaluation of cholesterol metabolism genes and cholesterol metabolism patterns have the potential to predict the outcome of immunotherapy and guide therapeutic strategies.

TMB analysis showed that the number of base mutations was significantly higher in patients in the low-risk group than in the high-risk group, an observation that was strongly associated with a better prognosis for patients in the low-risk group. This finding was associated with advantages in chemotherapy response and immune response in patients with high TMB. A study by Park *et al.*^33^ noted that small-cell lung cancer patients with higher than median TMB had significantly prolonged progression-free survival and overall survival after concurrent radiotherapy. In the field of GC, the results of Li *et al.*^34^ similarly showed that patients with high TMB were positively associated with the efficacy of neoadjuvant chemotherapy, and the disease-free survival of these patients was significantly higher than that of patients with low TMB. In addition, we analyzed the sensitivity of chemotherapy drugs and found that patients in the low-risk group had lower drug-sensitive concentrations of chemotherapy drugs such as bleomycin, camptothecin, cisplatin, and doxorubicin. This result suggests that we should pay extra attention to the response of gastric cancer patients in the high- and low-risk groups to different chemotherapy regimens, which can be used as a basis for the development of individualized treatment strategies.

Through single-cell analysis, we examined the differences in cell types within the tumor microenvironment between high-risk and low-risk groups, comparing DKK1, F5, GFRA1, GPC3, and HABP2 expression in gastric cancer (GC) tissues with normal tissues, as well as between *Helicobacter pylori*-infected and uninfected GC tissues. DKK1 may influence the immune system's ability to recognize and eliminate tumor cells by regulating the activity of immunosuppressive cells, such as Treg cells and tumor-associated macrophages (TAMs)^35^. GFRA1 plays a role in the growth and repair of the nervous system, and its aberrant expression may be associated with cancer cell proliferation and migration, indicating its potential as an immunotherapy target^36^. Furthermore, single-cell analysis revealed distinct expression patterns of coagulation-related genes in different immune cells, which could help identify molecular markers associated with patient prognosis in a clinical setting. High F5 gene expression, for instance, may indicate an increased risk of thrombosis, a critical prognostic factor in cancer patients^37^. GPC3, known as a tumor marker in certain cancers (e.g., hepatocellular carcinoma), may serve a similar role in gastric cancer, suggesting that high expression in specific cell types could aid in disease prognosis^38^. Additionally, tumor immune single-cell analysis can identify immune cell subpopulations responsive to immunotherapy. For example, HABP2 expression in certain immune cells may be related to immune evasion or anti-tumor immune responses. In-depth analysis of these genes' expression in various immune cell types could provide valuable insights into optimizing immunotherapy strategies, such as immune checkpoint inhibitors (e.g., PD-1 inhibitors)^39^. In conclusion, tumor immune single-cell analysis not only uncovers the expression patterns of coagulation-related genes across different cell types in gastric cancer tissues but also offers valuable information for clinical applications such as personalized therapy, prognosis evaluation, and the optimization of immunotherapy.

Increased tumor mutation burden may promote the expression of tumor antigens, thereby enhancing the body's immune response and thus improving the prognosis of patients^40^-^42^. In the present study, we compared the differences in the immune microenvironment between GC patients in the high-score group and the low-score group, and the results showed that the content of T-cell follicular helper cells and activated CD4+ memory T cells in the low-score group was significantly higher than that in the high-score group. This suggests that the number of critical CD4+ T cells was higher in the low-score group, and CD4+ T cells play an important role in GC immunotherapy. The study by Gao *et al.*^43^ noted that the percentage of peripheral blood CD4+ T cells expressing PD-1 was significantly higher in treatment-effective advanced GC patients than in treatment-ineffective patients, further emphasizing the key role of CD4+ T cells in immunotherapy efficacy. Our high- and low-risk groupings were effective in differentiating immunotherapy-sensitive patients and augur well for a more personalized immunotherapy regimen based on the patient's immune microenvironmental status.

Our study showed that prognostic coagulation-related genes screened by Lasso regression could predict the prognosis of GC patients more accurately. Based on the risk score calculated from prognostic coagulation-related genes, GC patients could be significantly divided into two groups: high TMB and low TMB. In addition to this, we further analyzed the characteristics of tumor immune infiltration in patients in the high- and low-risk groups. The differences in TMB and immune infiltration characteristics between the high and low-risk groups can help the selection of chemotherapy and immunotherapy regimens for patients. Therefore, in addition to screening new prognostic biomarkers for GC, our study also provides a theoretical basis for individualized chemotherapy and immunotherapy for GC patients.

## Supplementary Material

Supplementary figures and tables.
