# Supplementary material for: Clinical significance and immune landscape analyses of the coagulation-related gene signatures in gastric cancer
Source: J Cancer. 2025 Mar 3;16(6):1971–86. doi: 10.7150/jca.104221 (PMC11905418; doi:10.7150/jca.104221)
Supplement: Supplementary file 1 — Supplementary figures and tables. [file jcav16p1971s1.zip › Supplementary materials/Supplementary figures.pdf]

Figure legends

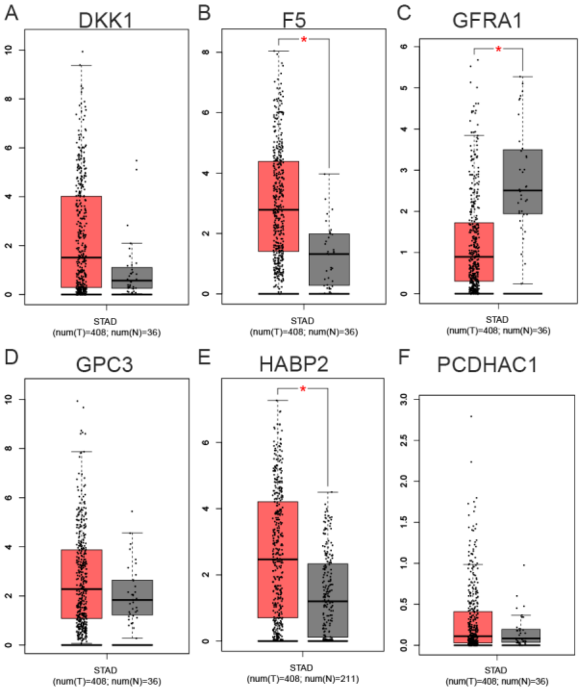

Supplementary Figure S1: Differential expression of DKK1, F5, GPC3, HABP2, PCDHAC1, GFRA1 in GC tissue and normal tissue.

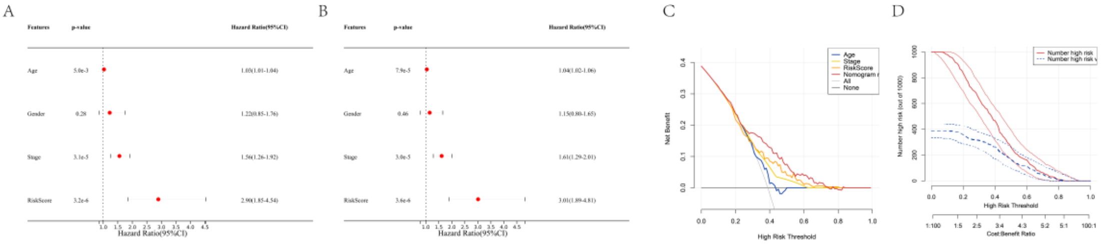

Supplementary Figure S2: (A) Univariate COX regression analysis; (B) Multivariate COX regression analysis; (C) Decision curves; (D) Clinical impact curves.
